# Unraveling assumptions about clinical relevance in patient-reported outcome data: A qualitative study on perspectives of different interest groups

**DOI:** 10.1007/s00520-026-10387-6

**Published:** 2026-02-02

**Authors:** Ines S. Rathgeber, Daniela Krepper, Lisa M. Wintner, Johannes M. Giesinger, Monika Sztankay

**Affiliations:** Department of Psychiatry, Psychotherapy, Psychosomatics and Medical Psychology, University Hospital of Psychiatry II, Medical University of Innsbruck, Anichstrasse 35, 6020 Innsbruck, Austria

**Keywords:** Patient-reported outcomes, Quality of life, Clinical relevance, Clinical importance, Meaningful interpretation, Clinical oncology

## Abstract

**Purpose:**

The interpretation of patient-reported outcomes (PROs) in oncology research lacks a shared understanding of clinical relevance among interest groups. Terms like *minimal important differences* and *clinically meaningful change* aim to aid interpretation but remain inconsistently conceptualized. This study explores interest-holders’ perspectives to support setting-specific definitions of clinical relevance in PRO research.

**Methods:**

An online survey was distributed via international networks to multi-professional interest-holders. Responses to an open-ended question regarding participants’ understanding of clinical relevance were analyzed using qualitative content analysis to identify recurring themes and patterns within the quotes.

**Results:**

The survey included 92 participants: clinical practitioners (38.5%), academic researchers (47.3%), industry researchers (8.8%), and patients/patient representatives (rep.) (5.5%). Five clusters emerged reflecting facets of clinical relevance: (a) patient value (e.g., impact on well-being), (b) practical implications (e.g., treatment changes), (c) external criteria (e.g., physiological changes), (d) statistical approaches (e.g., 10% difference), and (e) proxy value (e.g., physician’s perspective). Practitioners primarily focused on patient value (55.6%), while academic researchers showed a similar distribution but with greater variance across clusters. In contrast, industry researchers more frequently emphasized external criteria (20.0%) and proxy value (20.0%) compared to other groups. *Patient value* and *statistical approaches* were not mentioned by the same participants, nor were *external criteria* and *practical implications*.

**Conclusion:**

Conceptual understanding of clinical relevance varies by professional background, highlighting its multifaceted nature. These findings, identifying distinct conceptual clusters across interest groups, provide a foundation for developing harmonized, context-specific definitions of clinical relevance in PRO research.

**Supplementary Information:**

The online version contains supplementary material available at 10.1007/s00520-026-10387-6.

## Background

Patient-reported outcomes (PROs) have become a cornerstone in evaluating the efficacy and impact of oncology treatments from the patient’s perspective, are a standardized component for high-quality cancer clinical trials [1, 2], and are also increasingly being integrated into clinical practice [3, 4]. The US Food and Drug Administration (FDA) has endorsed incorporating PRO measures (PROMs) into the benefit-risk assessment in cancer trials and has published guidance on their assessment [5].

The interpretation of PROs in oncology research and practice is a multifaceted issue. A statistically significant difference may not be clinically relevant, as it provides no insight into the relevance for the patient, or, for instance, the necessity for changes in treatment [6, 7]. When talking about clinical relevance, the scientific community refers to terms like “minimal important differences” (MIDs) or “clinically meaningful changes”. However, there is a lack of consensus on definitions, terminology, and methodology [8–10].

Definitions of MIDs available in the literature shed light on considerations about the nature of clinical relevance in the field. One of the most influential definitions, according to King and colleagues [11] comes from Jaeschke et al. [12] who define “the minimal clinically important difference (MCID) […] as the smallest difference in score in the domain of interest which patients perceive as beneficial and which would mandate, in the absence of troublesome side effects and excessive cost, a change in the patient's management.” Schünemann and colleagues [13] provide the following definition: “We now define the MID as the smallest difference in score in the outcome of interest that informed patients or informed proxies perceive as important, either beneficial or harmful, and which would lead the patient or clinician to consider a change in the management”.

In research practice though, clinical relevance is usually defined implicitly by the choice of method to determine an MID, e.g., by the use of an external indicator, called ‘anchor’: In the literature, various approaches to the determination of anchor criteria can be found [6]. The anchor can be, for example, physician-reported, e.g., the performance status (e.g., [14–17]), a patient-reported rating of change [18] or supportive care needs [19, 20]. A combination of approaches is recommended [21] and frequently applied [22]. However, the approaches commonly used often do not align well with the established definitions of clinical relevance mentioned above, e.g., the change in management or treatment is not depicted in anchor-based approaches. This indicates a gap between existing definitions and research practice, alongside a lack of comprehensive, standardized definitions to which theory and research practice can refer.

Without an explicit framework, variability in understanding and conceptualizing clinical relevance may lead to inconsistencies in research, its interpretation, and clinical application. For instance, what is considered clinically relevant from a research perspective may not match a clinician’s or a patient’s perception or the expectations from regulatory authorities [23]. Therefore, it may be useful to define clinical relevance differently depending on the application (e.g., drug approval, shared decision-making, interpretation of trial results) in a methodologically standardized way. Only by reaching a consensus on the methods for determining clinical relevance can clinical studies be properly designed, interpreted, and evaluated, and their results successfully implemented (see also [24]). Those differentiations should happen explicitly with a clear and explicit underlying concept of what is clinically relevant, decisive, and meaningful for the respective settings.

This study addresses this gap by analyzing qualitative data regarding the understanding and conceptualization of clinical relevance among various interest groups in PRO research. The aims are to (1) investigate the understanding of the concept of clinical relevance in PRO research in oncology and (2) compare different interest groups’ perspectives on the latter.

## Methods

### Study design and participants

The data reported was assessed for the European Organisation for Research Treatment of Cancer (EORTC), Quality of Life Group (QLG) project aiming to develop an Interpretation Guideline for the EORTC PRO measures (grant number: 006–2021). Phase I of the project comprised an online survey conducted between November 2021 and September 2022, aiming to assess how different interest-holders interpret PRO data collected with EORTC measures. The extended survey period reflects multiple recruitment waves, which were necessary to ensure adequate disciplinary representation.

Besides questions targeting experiences and needs regarding interpretation approaches, the survey included an open-ended question encouraging participants to provide their understanding of the concept of clinical relevance: “Please explain in your own words, what clinical relevance in the context of PRO data means to you.” The survey was distributed to the project’s expert panel, and additional interest-holders were recruited via the EORTC QLG and the EORTC Disease Oriented Groups (DOGs) and via purposeful snowball sampling within the pharmaceutical industry.

The nature of the online survey ensured that researchers and respondents remained unknown to each other, with no prior relationship. Although recruitment occurred via professional networks (e.g., EORTC QLG), participation was voluntary and anonymous. The researchers, psychologists experienced in PRO research, were aware of their own science-informed assumptions about clinical relevance and used reflexive discussions to minimize potential bias.

### Data analysis

Qualitative content analysis was applied to participants’ quotes, which is an inter-subjectively comprehensible and rule-based method for analyzing qualitative material [25, 26]. Category formation followed an inductive approach in line with the conventional category development as described by Hsieh and Shannon [27]. This allows information to be gathered directly from study participants without imposing pre-determined categories, thus enabling an exploratory approach to a topic.

The coding process was a five-step procedure consisting of (1) familiarization with the material; (2) initial code development and creation of a coding scheme of recurring themes and patterns (performed by IR); (3) piloting of a set of example quotes (*N* = 10) and fine-tuning decision rules, ensuring replicability across coders (IR, DK); (4) application of the final coding scheme by double coding of the entire material (IR, DK); (5) discussion of non-agreements in the allocation of codes in consensus meetings and resolvement through joint refinement of the coding framework (IR, DK); and (6) consultation of a third person in case of remaining discrepancies (MS). Through joint discussion, a consensus agreement was achieved for all remaining cases. It was allowed to assign an individual quote to more than one code.

In the next step, codes were aggregated into overarching content clusters (based on thematic congruence). This step helps to arrange and structure the multitude of codes, allowing the key themes to be extracted from the data. This process led to the final assignment of the individual quotes to one or more content clusters, depending on the affiliation of the respective codes assigned in the previous step. Since it was allowed to assign quotes to more than one code, quotes could also be part of more than one cluster.

Analyses were conducted targeting the frequency of allocated clusters as well as the overlap in allocations of each of the clusters in individual quotes. Additionally, interest groups were compared concerning the identified clusters in their respective quotes.

NVivo Version 14 (2023) was used for qualitative data analysis. For subsequent quantitative analysis (e.g., frequencies), SPSS Version 29 (2023) was applied. The reporting in this manuscript followed the 32-item COREQ (Consolidated Criteria for Reporting Qualitative Research) checklist [28].

## Results

### Participants

A total of 92 individuals answered the open-ended question concerning their understanding of the concept of clinical relevance (complete answers are provided in supplementary material, A).

Participants were mainly between 30–45 (36.3%) and 46–60 (39.6%) years old. The sample was well balanced with regard to participants’ sex (52.2% women). Participants were from various backgrounds: Practitioners without research background (*n* = 35, 38.5%), academic researchers (*n* = 43, 47.3%), researchers with an industry background (*n* = 8, 8.8%), and patients (rep.) (*n* = 5, 5.5%), information missing (*n* = 1). Participants identifying as both practitioners and researchers were included in the researcher group, assuming their research background would have a major impact on their perception of PRO clinical relevance. Of those reporting their field of specialization (data missing, *n* = 1), the majority (64.1%) were in oncology. Table 1 summarizes participant characteristics: 83.7% had experience with EORTC PRO data in clinical research, 43.3% in practice, and among those reporting years of experience (*n* missing = 16), 75.1% had more than six years of research experience with EORTC PRO data.

**Table 1:** Characteristics of the survey participants (*N* = 92)

Variable		***N***	Valid %
Sex	Female	48	52.2
	Male	44	47.8
	Other	0	0.0
Age	< 30	6	6.6
	30–45	33	36.3
	46–60	36	39.6
	> 60	16	17.6
	Missing	1	
Professional background	Clinical practitioners	35	38.5
	Academic researchers	43	47.3
	Researchers from industry	8	8.8
	Patients/patient rep	5	5.5
	Missing	1	
Field of specialization*	Oncology	50	64.1
	PROs/quality of life	6	7.7
	Psycho-oncology	4	5.1
	Surgery	3	3.9
	Patient advocacy	3	3.9
	Clinical trials	2	2.6
	Other	10	12.8
	Missing	14	
Experience with EORTC PRO data in clinical practice	Yes	36	43.4
	No	47	56.6
	Missing	9	
Experience with EORTC PRO data in research	Yes	77	83.7
	No	15	16.3
Years of experience using EORTC data in research	< 2	7	9.2
	2–5	12	15.8
	6–10	22	29.0
	11–20	19	25.0
	> 20	16	21.1
	Missing	16	

*Multiple answers possible

### Content clusters of the concept of clinical relevance in PROs

Within the qualitative content analysis, the 92 quotes were assigned 130 times to 15 unique codes, with 33 (35.9%) of the quotes being assigned to more than one code.

Codes were grouped into five key content clusters. These clusters revealed different main approaches to the concept of clinical relevance: *a. patient value*,* b. practical implications*,* c. reference to external criteria*, *d. a statistical approach*,* e. proxy value.*

Table 2 summarizes the results of the content analysis, including representative quotes from the participants for each code. In some cases, more than one code from a single quote was assigned to the same cluster, which is why the total number of cluster allocations (*N* = 104) is lower than the total number of code allocations (*N* = 130). Detailed code and cluster frequencies are provided in supplementary material B.

**Table 2:** Clusters and codes representing participants’ perspectives regarding the concept of clinical relevance (*N* = 92 quotes)

Codes (*N* = 15)	Code description	Example quote (quote-number)	Clusters (***N*** = 5) *Total allocations N = 104*	Cluster description
A.1 Meaningful for patient	*The importance for the health status of the patient him-/herself is at the centre*	“What patients find relevant—moving around, less pain, being able to travel” (Q6)	A. Patient value (***n*** = 49)	*Participants express that clinical relevance in the context of PRO data means that there is relevance for the patient*
A.2 Noticeable for patient	*The mere perception of a difference by the patient is at the centre*	“A change in patient report of scores that is perceptible to him/her” (Q31)		
A.3 Impact on patient	*If no category of the above applies but any kind of impact on the patient is described*	“Does it directly affect the patient ie make it better or worse” (Q44)		
B.1 Action must be taken	*The need for action is described by* *explicit mention of a required action* *e.g., change of medication, decision making *etc.	“when the difference is so important that I change treatment for this reason” (Q56)	B. Practical implications (***n*** = 32)	*Participants express that clinical relevance in the context of PRO data means that there is an impact on clinical practice*
B.2 Importance for clinical practice	*Impact on requirements for clinical action in some other form* *e.g., results are applicable for clinical practice (without explicit need for action)*	“How relevant the results are to the clinical indication” (Q65)		
B.3 Clinical importance	*Only a clinical importance is described - without designated reference to implications for practice or medical/physical values*	“Whether change is meaningful in a clinical context” (Q68)		
C.1 Reference to clinical values	*There is a correlation to a measurable ****clinical**** value*	“Clinical relevance ie: either associated with ECOG change or minimum G2 toxicity (affecting QoL)” (Q73)	C. Reference to external criteria (***n*** = 9)	*Participants express that clinical relevance in the context of PRO data means that objective external criteria are met*
C.2 Reference to objectively measurable criteria	*Emphasis on something that is clearly or objectively measurable*	“[…] or costs/resurses [sic] are measurably different” (Q11) “an actual, (physically) observable difference between two scores” (Q78)		
D.1 Difference to control group	*An identified difference to control group*	“it gives information about the individual patient in relation to the group of same patients” (Q79)	D. Statistical approach (***n*** = 7)	*Participants express that clinical relevance in the context of PRO data means that a statistical requirement is met*
D.2 Statistical parameters	*e.g., Osoba rule, MIDs*	“10% change” (Q81)		
E.1 Meaningful for health care professionals or other proxies	*The importance for proxy him-/herself is at the centre*	“PRO data are clinically relevant when they assess issues of clinical importance for patients as well as for HCPs[…]” (Q26)	E. Proxy value (***n*** = 7)	*Participants express that clinical relevance in the context of PRO data means relevance for any individual associated with the patient, beyond the patient themselves*
E.2 Noticeable for health care professionals or other proxies	*The pure noticing of a difference by the proxy is at the centre*	“A clinically relevant difference is a difference that is noticed/mentioned by […] proxies.” (Q40)		
X.1 Differentiation to statistical significance (*n* = 13)	*Clinical relevance is explained in contrast to statistical significance*	“[…], this does not mean only the significant statistical changes in scores” (Q22)	No thematic congruence to clusters	
X.2 Any change (*n* = 1)		“any change in a pro measurement” (Q84)		
X.3 Not applicable (*n* = 6)	*Everything that does not answer the question*	“Whether the difference or change in PRO outcome is also clinically relevant.” (Q90)		

More than half of the quotes (49, 53.3%) could be assigned to the cluster *a. patient value*, which focuses on the value patients themselves attach to the change or difference in score. Descriptions ranged from *a *“meaningful impact on [the] quality of life” (Q3) to something that is“noticeable for the patient” (Q23) or a mere “impact on patients” (Q43). The common idea of quotes that are allocated to this cluster is that the patient perspective is central to assessing clinical relevance.

Cluster *b. practical implications* was assigned to 32 quotes (34.8%). Participants stated that PROs are clinically relevant if either a specific action is required or, more generally, there is importance for clinical practice, such as treatment changes, care adjustments, or the need for further investigation. One participant described it as “a difference that may lead to a clinical action/decision: choose a special intervention, change or continue symptom relief” (Q53).

Cluster *c. reference to external criteria* was identified in 9 (9.8%) of the quotes. Participants referred to clinical values, considering PRO changes clinically relevant when linked to other outcomes, such as a change in CTCAE Grade 2 toxicity or physical functioning. Quotes in this cluster also emphasized determination of clinical relevance by objectively measurable criteria, e.g., by an “actual (physically) observable difference between two scores” (Q78) or “costs/resurces [sic] [that] are measurably different” (Q11).

Another perspective on clinical relevance is encompassed in cluster *d. statistical approach*, to which 7 (7.6%) of the quotes were assigned. Quotes emphasized the difference to a control group t, stating, e.g., clinical relevance in PROs “gives information about the individual patient in relation to the group of same patients” (Q79). Participants also referred to statistical parameters of clinical relevance in PROs, such as a 10% change in scores.

Cluster *d. proxy value* was assigned to 7 (7.6%) of the quotes, describing clinical relevance as something that is evaluated by or with physicians, other health care professionals (HCPs), or any other individuals other than the patient themselves, considering something clinically relevant if it is meaningful to or noticed by patients, proxies, or both.

Three codes were not assigned to clusters: *Differentiation to statistical significance* was assigned to 13 (14.1%) of the quotes, sometimes as the sole explanation for clinical relevance, lacking conceptual content for clustering. One quote was coded as *any change* (1, 1.1%), stating that any change in the values was clinically relevant (Q84)—noted but not clustered or further analyzed. The code *not applicable* was assigned to 6 (6.5%) of the quotes as they did not answer the question. Those quotes were not excluded for insights into question comprehension, which also provides important information.

### Overlaps in the allocation of clusters

Clusters with a low rate of allocation overlap (i.e., allocation to two clusters within the same quote) could represent distinct perspectives on the concept, while high overlap suggests conceptual links. *Patient value* frequently co-occurred with other clusters: In 10 (20.4%) of the quotes that were assigned to *patient value*, *also practical implications* were mentioned. 100% of the quotes that were assigned to *proxy value* also contained the cluster *patient value*.

No co-occurrence was observed for *patient value* with *statistical approach* or *reference to external criteria* with *practical implications* (see supplementary material C and D for detailed cluster relationships).

### Comparison of interest groups

Table 3 shows the five main content clusters of clinical relevance identified within the participants’ quotes according to professional background. Due to larger sample sizes, the main comparison can be made between the group of practitioners (*n* = 34) and the group of academic researchers (*n* = 43). However, to allow for indications of different subgroups, researchers from industry (*n* = 8) as well as patients and patient representatives (*n* = 5) are listed and analyzed individually. Cluster allocations are presented as proportions relative to the number of quotes within each group.

**Table 3:** Cross table cluster—interest-holders grouped

		Practitioner	Academic researcher	Industry researchers	Patient (rep.)	Total cluster allocations
Patient value	*n*	20	22	3	3	48
	% of group*	57.1%	51.2%	37.5%	60.0%	
Practical implications	*n*	10	19	3	0	32
	% of group	28.6%	44.2%	37.5%	0.0%	
Reference to external criteria	*n*	3	3	2	1	9
	% of group	8.6%	7.0%	25.0%	20.0%	
Statistical approach	*n*	1	5	0	1	7
	% of group	2.9%	11.6%	0.0%	20.0%	
Proxy value	*n*	2	3	2	0	7
	% of group	5.7%	7.0%	25.0%	0.0%	
Total (*n* per group)		35	43	8	5	91**/103

*Proportion of cluster allocation relative number of quotes per group

**Information on professional background missing, *n* = 1

The cross-table shows that all groups provided quotes that were assigned to the *patient value* cluster, most frequently patient representatives (60.0%) and physicians (57.1%). Only among researchers from industry *patient value* appeared in less than half (37.5%) of the quotes.

While both quotes of practitioners and academic researchers can be assigned most frequently to the cluster *patient value*, notable differences arise for *practical implications*. Academic researchers mention content attributable to this cluster in 44.2% of quotes versus 28.6% for practitioners. The patient (rep.) responses do not refer to *practical implications* at all. A similar pattern is observed for the *statistical approach*, identified in 11.6% of academic researcher quotes, while allocated significantly less often, in 2.9% of practitioner quotes.

A total of 13 participants (14.1%) mentioned the distinction from statistical significance in their description of clinical relevance. It was most brought up by academic researchers (*n* = 7, 16.3%), but also by practitioners (*n* = 5, 14.3%) and researchers from industry (*n* = 1, 12.5%).

**Fig. 1:**
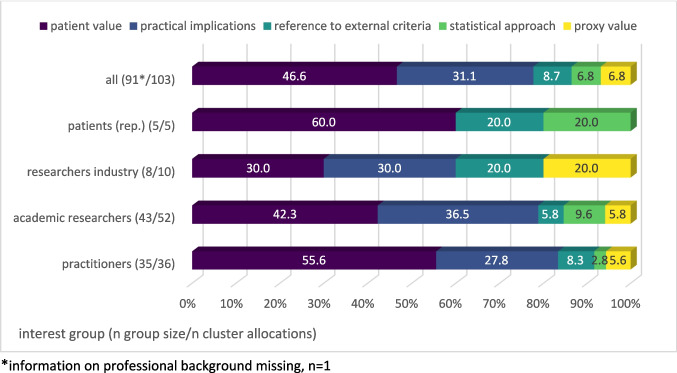
Differences in content clusters according to interest groups: Proportions of specific cluster allocations relative to the number of all cluster allocations within the groups’ quotes

As one quote could be allocated to multiple clusters, the number of cluster allocations per group differs from the number of quotes. Figure 1 depicts the distribution of each group’s cluster allocations across the five key clusters, with proportions relative to the total allocations within a group’s quotes (100%).

Both researchers from industry and patient (rep.) show another pattern than the overall group, practitioners, and academics. While the quotes of researchers from the industry, unlike those of the other groups, were less often allocated to the *patient value* cluster, the clusters *reference to external criteria* and *proxy value* are, with both 25% of the quotes, assigned most frequently compared to the other groups.

The majority of the patients’ (rep.) quotes (60.0%) can be allocated to the *patient value* cluster. There is little variety in the content clusters of the quotes by patients and patient representatives in contrast to the other interest groups. *Practical implications* and *proxy value* cannot be identified at all in the quotes from patients and patient representatives.

## Discussion

Considering the ongoing discussion about the meaningful interpretation of PRO scores in oncology, this study provides valuable insights into the multifaceted nature of clinical relevance in the context of PROs by analyzing the perspectives of various interest holders. As highlighted in prior research [11, 29], the concept of clinical relevance lacks harmonization in terminology and methodology. This study elucidates the assumptions underlying clinical relevance among different interest groups.

### Identified clusters and allocation overlaps

The qualitative analysis identified five key clusters in participants’ understanding of clinical relevance: (a) *patient value*, (b) *practical implications*, (c) *reference to external criteria*, (d) *statistical approach*, and (e) *proxy value*. Among these, *patient value* was identified most often, in 53.3% of the quotes. Notably, *patient value* and *practical implications* were often mentioned together, suggesting that they are part of a joint perspective on clinical relevance. By contrast, the complete absence of overlap between *patient value* and *statistical approach* might reflect divergent perspectives on what constitutes meaningful change: experiential judgments grounded in what patients perceive as relevant versus technically defined, distribution-based thresholds (e.g., a 10% change). Respondents who based their answers on statistical criteria did not reference patient perceptions, suggesting that these approaches are often applied as technical tools, with only limited connection to patients’ lived experience. Similarly, *practical implications* and *reference to external criteria* seem to represent distinct perspectives, the former on actionable insights for clinical practice, the latter on trial efficacy thresholds.

The content clusters identified herein are in large parts consistent with the ideas of clinical relevance behind commonly used approaches for the determination of MIDs (for an overview of approaches, see [6]): *Patient value* (anchored by perceived change, e.g., transition items), *reference to external criteria* (anchored by, e.g., weight loss), *statistical approach* (distribution-based approaches), and *proxy value* (anchored by, e.g., physician-rated performance change). They also echo Jaeschke et al. [12] and Schünemann et al. [13] definitions, combining perceived importance (*patient value*) with management changes (*practical implications*). Yet the patient-proxy linkage highlights a conceptual gap: while proxies are theoretically tied to patient views, MID methods frequently operationalize them independently, potentially overlooking these assumptions. Likewise, the patient-statistical disconnect underscores how quantitative rules may prioritize measurement over patient-centered meaning. However, *practical implications*, a frequent cluster mirroring common MID definitions [12, 13, 30], are underrepresented in most prominent approaches for determining a MID relying on external criteria, statistics, or proxies [6, 29]. *Patient value* and *proxy value* showed strong co-occurrence, with no participant mentioning proxy value alone. This pattern suggests epistemic dependencies, where practitioners and other proxies frame their judgments within assumed patient priorities, e.g., inferring patient importance rather than treating proxy views as fully independent. Such embedding aligns with patient-centered rhetoric but contrasts with research practice, where proxy-based anchors (e.g., physician-rated performance) are often used standalone [6, 29]. Moreover, 14.1% of quotes were assigned to the code *differentiation from statistical significance*, indicating that some respondents found it easier to express their perception of clinical relevance by contrasting it with statistical significance rather than providing a positive definition.

These patterns call for a harmonized PRO framework that explicitly integrates patient value/practical implications with other clusters, clarifying when changes warrant management shifts, and probing why patient and statistical views diverge.

### Differences in interest groups

Assumptions concerning the concept of clinical relevance might differ regarding the professional backgrounds. Quotes of practitioners predominantly could be allocated to *patient value*, whereas those from industry researchers often showed aspects belonging to *proxy value* and *external criteria*, reflecting that they may have aspects in mind that are reflected in common anchor approaches for MIDs (e.g., physician-rated performance change) and therefore a stronger research-driven perspective. The fact that patient responses in our sample were not assigned to the *practical implications* cluster could be interpreted as reflecting that patients may not perceive PRO data as directly influencing treatment decisions. Within the quotes from academic researchers, a large variety of aspects referring to the defined clusters could be identified, likely due to their interdisciplinary backgrounds.

These differences underscore the challenges in defining and applying clinical relevance in PRO research. For example, clinicians focus on patient care and practical implications, while researchers and industry professionals are (also) concerned with endpoints that measure the efficacy of treatments. These differences in how clinical relevance is framed across interest groups point to a lack of consensus and potential misunderstandings, e.g., in the cross-disciplinary interpretation of research results.

### Strengths and limitations

A key strength of this study is its relatively large sample size, allowing for a detailed analysis of participant assumptions regarding PROs and clinical relevance. However, participant groups were unevenly distributed, with most respondents from research backgrounds (56%), a smaller proportion of practitioners (38%), and only a few patient representatives (5%). Consequently, also due to the sampling strategy, the findings primarily reflect the perspectives of experts, providing limited insight into patient viewpoints. As a result, also the concept of *patient value* in this study is primarily based on the interpretation of the members of participating interest groups rather than direct patient data. Given the very small number of patient respondents, no firm hypotheses about their response pattern can be drawn.

The study was exploratory in nature, and thematic saturation was not formally assessed. Additionally, the study’s reliance on short written quotes for evaluation limits the depth of analysis. Incorporating case examples through interviews or focus groups could provide more context and richer insights. Further qualitative research is needed to better clarify the concept of clinical relevance and its underpinnings. Specifically, exploring patient perspectives would offer valuable insights, as their views are critical for defining clinical relevance. Comparing patient and expert perspectives could also highlight shared and divergent views, offering a more comprehensive understanding of the concept.

### Implications and further directions

The identified clusters provide empirically grounded insights that can inform ongoing international standardization efforts, also addressing PRO interpretation such as the recommendations of the Setting International Standards in Analyzing Patient-Reported Outcomes and Quality of Life Endpoints Data (SISAQOL-IMI) Consortium [31] and national guidelines from the German “Institut für Qualität und Wirtschaftlichkeit im Gesundheitswesen (IQWIG)” [9]. The prominence of *patient value*, often co-occurring with *practical implications*, aligns with SISAQOL-IMI’s emphasis on context-sensitive and patient-centered interpretation, while also highlighting opportunities to further clarify when observed PRO changes should prompt clinical action. At the same time, the lack of overlap between *patient value* and *statistical approach* underscores the need to better integrate experiential perspectives into quantitatively defined thresholds. Similarly, the reliance on externally anchored and distribution-based approaches within existing IQWiG guidance mirrors less frequent clusters observed in this study, reinforcing the importance of transparent anchor selection and caution against reliance on single criteria. Looking ahead, an important question for future harmonized frameworks is how clinical relevance can be operationalized beyond previous approaches by explicitly incorporating practical implications. Greater clarity and harmonization in defining clinical relevance, through explicit reporting, combined anchors, and validation of proxy judgments against patient priorities, may help prevent misinterpretation and the uncritical application of predefined thresholds, supporting more consistent, meaningful, and patient-relevant use of PRO data across oncology research and practice.

## Conclusion

This study identified five key content clusters regarding participants’ assumptions about clinical relevance in PROs and reveals significant variation in how interest-holders define the concept. *Patient value* emerges as the most identified cluster, often linked to *practical implications*. While some clusters align with existing approaches to determining MIDs, aspects like practical implications remain underrepresented in traditional methodologies.

To enhance understanding of clinical relevance, it is vital to validate these findings with a more diverse sample, including patient perspectives. Greater clarity and harmonization in defining clinical relevance for different settings will ensure PRO scores are interpreted consistently and meaningfully, ultimately improving patient care and supportive oncology interventions.

## Supplementary Information

Below is the link to the electronic supplementary material.

ESM1 (DOCX.109 KB)

## Data Availability

The author confirms that data generated or analyzed during this study are included in the supplementary files of this published article.
