# Supplementary material for: Unraveling assumptions about clinical relevance in patient-reported outcome data: A qualitative study on perspectives of different interest groups
Source: Support Care Cancer. 2026 Feb 2;34(2):155. doi: 10.1007/s00520-026-10387-6 (PMC12864281; doi:10.1007/s00520-026-10387-6)
Supplement: Supplementary file 1 — (DOCX.109 KB) [file 520_2026_10387_MOESM1_ESM.docx]

**Supplementary material**

**Unraveling assumptions about clinical relevance in patient-reported outcome data**

**A qualitative study on stakeholders’ perspectives**

*Ines S Rathgeber, Daniela Krepper, Lisa M Wintner, Johannes M Giesinger, Monika Sztankay*

[*ines.rathgeber@i-med.ac.at*](mailto:ines.rathgeber@i-med.ac.at)

*University Hospital of Psychiatry II, Medical University of Innsbruck, Innsbruck, Anichstrasse 35, 6020 Innsbruck, Austria*

***A. Overview Codes and References***

| **Codes** | Quotes with coded text | Quote ID |
| --- | --- | --- |
| **Patient value** |  |  |
| Meaningful for patient | Clinical relevance is the level of severity (or the change to that level) that matters to the patient and is likely to prompt a further discussion, order investigations or change medications. | Q1 |
|  | it means that the change in QoL is important from patient perspective - it is important point to dicuss when you have few strategies but different QoL results | Q2 |
|  | Any change is sufficiently large that the patient would be aware of it and feel that it had a meaningful impact on their quality of life or ability to do something (for better or worse) | Q3 |
|  | The change is scores that is meaningful to patients. | Q4 |
|  | Clinical relevance reflects if the change over time is meaningful for a patient. A change can be statistically significant (particularly when the sample is sufficiently large), but that doesn't mean the change is also clinically relevant. In my opinion, all PRO results should be interpreted in terms of both a statistical and clinical relevance. | Q5 |
|  | What patients find relevant - moving around, less pain, being able to travel | Q6 |
|  | What it means to an individual patient | Q7 |
|  | at patient-level it means that a change is considered meaningful by a patient (or can at least be perceived); at group-level it means that a difference is relevant for medical decision making etc | Q8 |
|  | That the results make a difference for the patient | Q9 |
|  | Clinical relevance is a measurable improvement or worsening of a symptom which is perceived by patients to effect their quality of life and ability to function as part of "normal daily life." | Q10 |
|  | A value /change of value which has an relevant impact on the patients well-being / health system / resurses = the patient can feel the difference, or costs / resurses are measurably different | Q11 |
|  | meaningful change for patients requesting change in treatment | Q12 |
|  | E.g. a difference or change is relevant/appreciable for the patient | Q13 |
|  | a change that is meaningful to the patient | Q14 |
|  | a meaningful change in quality of life data reported by patients | Q15 |
|  | The magnitude of the difference or change is relevant to/meaningful for the patient. | Q16 |
|  | Clinically relevant is a change that has important impact on the patient's quality of life. A difference of 1 in a score can become statistically significant if there are 1000 patients in the trial, but is may have NO clincal relevance at all! | Q17 |
|  | 1) An intervention is necessary to change a patients subjective well-being. 2) An intervention had an effect which is considered relevant to the pateint. Either way the patient may e.g. indicate a different score over time, but overall she/he feels stable and does not wish or need an intervention. | Q18 |
|  | a clinically important difference is valued against the data of patients themselves | Q19 |
|  | the results of a study are meaningful or not for several stakeholders | Q20 |
|  | a symptom or function ist c.r. if it affects the life/ health of a patient in a way that has relevance for him/ her | Q21 |
|  | clinical relevance should be based on the real reflection of patients' concerns. Hence, this does not mean only the significant statistical changes in scores | Q22 |
|  | Whether a change in score over time or difference between two scores represent a magnitude that is meaningful and noticeable for the patient. | Q23 |
|  | A différence that as a meanning in terme of patient appreciation or that imply a change in the way they are being taken care | Q24 |
|  | A score/score difference that indicates that patients have impairments/problems in their respective quality of life/domain | Q25 |
|  | PRO data are clinically relevant when they assess issues of clinical importance for patients as well as for HCPs/their institutions, e. g. when a change in a PRO score represents an acutal change in the health condition of a patient that influences his quality of life, the therapy or care etc. | Q26 |
|  | It is meaningful from patient's and/or physician's perspective. It could be interpreted by meaningful within-patient change or meaningful between-group difference. | Q27 |
|  | A difference can be statistically significant (in layman's terms, to large to likely be due to random chance), and yet not clinically relevant (e.g. the difference between the two groups begin compared is to small to be clinically important). Note: If a difference is large enough to mean a definition of clinically relevant, but it is not statistically significant, the difference is likely not clinically relevant. Clinical relevant difference is statistically significant difference that is large enough to be meaningful to patients and physicians. | Q28 |
| Noticeable for patients | Is the difference large enough to be noticeable to the average/most patients? | Q29 |
|  | an effect that can be experienced by the patient, i.e. depicts a difference/change of acutal clinical status | Q30 |
|  | A change in patient report of scores that is perceptible to him/her | Q31 |
|  | What difference between gruops or change over time that would be noticeable for the patients experiencing it | Q32 |
|  | Clinical relevance means is equivalent to the patient experiencing change. | Q33 |
|  | A change of the preception of the patient of his/her clinical status could be clinical relevant although could not translate to a statistical relevant result | Q34 |
|  | A change that will impact the patient's perception on his/her HRQoL and/or symptoms | Q35 |
|  | The change that is experiences by a patient or a group of patients. | Q36 |
|  | the patient **feels** worse/better | Q37 |
|  | in a big sample, a very small change/difference might be statistically significant. However, the same change might not make a real difference for patients. For example, a change of 5 points might be statistically significant, but if a patient experiences this change, he or she might not even notice it. | Q38 |
|  | Any change is sufficiently large that the patient would be aware of it and feel that it had a meaningful impact on their quality of life or ability to do something (for better or worse) | Q3 |
|  | at patient-level it means that a change is considered meaningful by a patient (or can at least be perceived); at group-level it means that a difference is relevant for medical decision making etc | Q8 |
|  | E.g. a difference or change is relevant/appreciable for the patient | Q13 |
|  | Whether a change in score over time or difference between two scores represent a magnitude that is meaningful and noticeable for the patient. | Q23 |
|  | That depends on whether for group differences, group mean change, or individual change. In broad terms, I would argue that relevance is either a noticeable (by an individual patient and/or their physician) difference or change in PRO data on an individual level, or a change/difference that is noticeable for a significant portion of patients in a group. | Q39 |
|  | A clinically relevant difference is a difference that is noticed/ mentioned by patients or their proxies. | Q40 |
|  | This is indeed a difficult problem. One generally understands the term so that a difference is clinically relevant if a patient or a clinicians thinks that the score has changed noticeably or significantly (not statistically significantly) for a single patient. However, I think that an intervention has also a clinically relevant effect if only every second patient profits from the intervention in a personally significant way; that is, I think that also half of the "individual" clinically relevant change is clinically meaningful | Q41 |
| Impact on patients | Clinical relevance is the real benefit that a patient derives from certain treatment, which can / can not be also statistically significat | Q42 |
|  | Improvement or deteriorating that has impact on patients | Q43 |
|  | This is indeed a difficult problem. One generally understands the term so that a difference is clinically relevant if a patient or a clinicians thinks that the score has changed noticeably or significantly (not statistically significantly) for a single patient. However, I think that an intervention has also a clinically relevant effect if only every second patient profits from the intervention in a personally significant way; that is, I think that also half of the "individual" clinically relevant change is clinically meaningful. | Q41 |
|  | does it directly affect the patient ie make it better or worse (so do we need to change treatments) | Q44 |
|  | Impact on quality of life or treatment decision making. | Q45 |
|  | when a change is relevant for a patient, when it actually impacts the life of a patient | Q46 |
|  | impact on patients lives | Q47 |
|  | How the patient in the real world is or will be affected. | Q48 |
|  | Priority was given to ensure the status of the subjects | Q49 |
| **Practical implications** |  |  |
| Action must be taken | clinically relevant is on the group level relevant for the planning of suportive care and erspective ressources and on the individual leven for "promunication" | Q50 |
|  | Has a practical importance in managing patients' disease | Q51 |
|  | that clinical action has to be taken | Q52 |
|  | A difference that may lead to a clinical action/decision: choose a special intervention, change or continue symptom relief | Q53 |
|  | I think that sometimes when you are interviewing patients you can get a feel for those that need more clinical intervention. It can open the door for discussion of more clinical problems that need investigation. | Q54 |
|  | The possibility of interaction with the patient and acting upon any symptom to relieve this. Regardless of how small the difference may be | Q55 |
|  | when the difference is so important that I change treatment for this reason | Q56 |
|  | Is the QoL score or the change in the score important to address? Maybe the QoL score between two groups is statistically different but still below the threshold of clinical relevance (not important enough to do something about it). | Q57 |
|  | does it directly affect the patient ie make it better or worse (so do we need to change treatments) | Q44 |
|  | Something worth taking note of and acting on (if things have worsened) | Q58 |
|  | It means that the patient has had a meaningful change in their clinical status, that in clinical practice may mandate a change in treatment, or in the context of clinical trials, may indicate a treatment effect. | Q59 |
|  | Priority was given to ensure the status of the subjects | Q49 |
|  | Clinical relevance is the level of severity (or the change to that level) that matters to the patient and is likely to prompt a further discussion, order investigations or change medications. | Q1 |
|  | it means that the change in QoL is important from patient perspective - it is important point to dicuss when you have few strategies but different QoL results | Q2 |
|  | meaningful change for patients requesting change in treatment | Q12 |
|  | 1) An intervention is necessary to change a patients subjective well-being. 2) An intervention had an effect which is considered relevant to the pateint. Either way the patient may e.g. indicate a different score over time, but overall she/he feels stable and does not wish or need an intervention. | Q18 |
|  | A différence that as a meanning in terme of patient appreciation or that imply a change in the way they are being taken care | Q24 |
|  | PRO data are clinically relevant when they assess issues of clinical importance for patients as well as for HCPs/their institutions, e. g. when a change in a PRO score represents an acutal change in the health condition of a patient that influences his quality of life, the therapy or care etc. | Q26 |
| Importance for clinical practice | Amount of change that matters clinically or practically | Q60 |
|  | if it really helps patients and care givers to improve their practise | Q61 |
|  | applicability of results in clinical practice | Q62 |
|  | Impact on quality of life or treatment decision making. | Q45 |
|  | whether the data is meaningful at bedside | Q63 |
|  | as correct as possible results for use in clinical practice an d guidelines. | Q64 |
|  | How relevant the results are to the clinical indication | Q65 |
|  | at patient-level it means that a change is considered meaningful by a patient (or can at least be perceived); at group-level it means that a difference is relevant for medical decision making etc | Q8 |
| Clinical importance | Clinical relevance refers to what is meaningful in real life. E.g an increase of lifespan of 2 weeks may be statistically significant but is not clinically relevant. | Q66 |
|  | Amount of change that matters clinically or practically | Q60 |
|  | Meaningful clinical change within a patient. | Q67 |
|  | a difference in PRO scores that is clinically meaningfull | Q68 |
|  | clinical meaninigsvoll | Q69 |
|  | Whether change is meaningful in a clinical context | Q70 |
|  | Clinically neaningful within-patient change or clinically meaningful between-group difference | Q71 |
| **Reference to**  **external criteria** |  |  |
| Reference to clinical values | Difference in PRO measure scores corresponds to clinical phenomenon (e.g., change in physical functioning, request for care). | Q72 |
|  | Clinical relevance ie: either associated with ECOG change or minimum G2 toxicity (affecting QoL) | Q73 |
|  | Improvement in PRO, even when "significant" is not equal to clinical relevance. Relevance means that the change is related a clinically meaningful outcome | Q74 |
|  | I believe that clinically relevant items that affect clinical data may be difficult to determine specifically in the data. There needs to be some type of measurement that specifically addresses the activity in order to be able to correlate the activity/data point to the PRO data. | Q75 |
|  | Clinical Relevance can help assess the scope of the effect of an intervention. For example, if patients are benefiting from an intervention via imaging, but their quality of life is significantly reduced, that is of clinical relevance and should be taken into account. | Q76 |
| Reference to objectively measurable criteria | the observed mean difference between groups demonstrate that on average, there is a real observable difference in that PRO domain between the two groups (e.g., less number of patients in control group can do a long walk relative to the treatment group) | Q77 |
|  | clinical relevance is whether there is an actual, (physically) observable difference between two scores | Q78 |
|  | Clinical relevance is a measurable improvement or worsening of a symptom which is perceived by patients to effect their quality of life and ability to function as part of "normal daily life." | Q10 |
|  | A value /change of value which has an relevant impact on the patients well-being / health system / resurses = the patient can feel the difference, or costs / resurses are measurably different | Q11 |
| **Statistical approach** |  |  |
| Difference to control group | it gives information about the individual patient in relation to the group of same patients | Q79 |
|  | Something that shows a difference on a patient group level. | Q80 |
|  | It means that the patient has had a meaningful change in their clinical status, that in clinical practice may mandate a change in treatment, or in the context of clinical trials, may indicate a treatment effect. | Q59 |
|  | the observed mean difference between groups demonstrate that on average, there is a real observable difference in that PRO domain between the two groups (e.g., less number of patients in control group can do a long walk relative to the treatment group) | Q77 |
| Statistical parameters | 10% change | Q81 |
|  | I refer you to the standard version, by osoba et al 1998 | Q82 |
|  | Scientifically validated MIDs | Q83 |
| **Proxy value** |  |  |
| Meaningful for HCPs or proxies | the results of a study are meaningful or not for several stakeholders | Q20 |
|  | It is meaningful from patient's and/or physician's perspective. It could be interpreted by meaningful within-patient change or meaningful between-group difference. | Q27 |
|  | A difference can be statistically significant (in layman's terms, to large to likely be due to random chance), and yet not clinically relevant (e.g. the difference between the two groups begin compared is to small to be clinically important). Note: If a difference is large enough to mean a definition of clinically relevant, but it is not statistically significant, the difference is likely not clinically relevant. Clinical relevant difference is statistically significant difference that is large enough to be meaningful to patients and physicians. | Q28 |
|  | PRO data are clinically relevant when they assess issues of clinical importance for patients as well as for HCPs/their institutions, e. g. when a change in a PRO score represents an acutal change in the health condition of a patient that influences his quality of life, the therapy or care etc. | Q26 |
| noticeability for HCPs or proxies | This is indeed a difficult problem. One generally understands the term so that a difference is clinically relevant if a patient or a clinicians thinks that the score has changed noticeably or significantly (not statistically significantly) for a single patient. However, I think that an intervention has also a clinically relevant effect if only every second patient profits from the intervention in a personally significant way; that is, I think that also half of the "individual" clinically relevant change is clinically meaningful. | Q41 |
|  | That depends on whether for group differences, group mean change, or individual change. In broad terms, I would argue that relevance is either a noticeable (by an individual patient and/or their physician) difference or change in PRO data on an individual level, or a change/difference that is noticeable for a significant portion of patients in a group. | Q39 |
|  | A clinically relevant difference is a difference that is noticed/ mentioned by patients or their proxies. | Q40 |
| **Remaining Codes** |  |  |
| Any change | any change in a pro measurement | Q84 |
| Differentiation to statistical significance | Improvement in PRO, even when "significant" is not equal to clinical relevance. Relevance means that the change is related a clinically meaningful outcome | Q74 |
|  | A change of the preception of the patient of his/her clinical status could be clinical relevant although could not translate to a statistical relevant result | Q34 |
|  | in a big sample, a very small change/difference might be statistically significant. However, the same change might not make a real difference for patients. For example, a change of 5 points might be statistically significant, but if a patient experiences this change, he or she might not even notice it. | Q38 |
|  | Statistically significant score differences do not necessarily translate into clinical significance | Q85 |
|  | Clinical relevance refers to what is meaningful in real life. E.g an increase of lifespan of 2 weeks may be statistically significant but is not clinically relevant. | Q64 |
|  | p value is not always significant, however there still might be a clinical significance Nelms, David W. M.D.; Vargas, H. David M.D.; Bedi, Ryan S. M.D.; Paruch, Jennifer L. M.D., M.S. When the p Value Doesn’t Cut It: The Fragility Index Applied to Randomized Controlled Trials in Colorectal Surgery, Diseases of the Colon & Rectum: February 2022 - Volume 65 - Issue 2 - p 276-283 | Q86 |
|  | Clinical relevance is the real benefit that a patient derives from certain treatment, which can / can not be also statistically significat | Q42 |
|  | Clinical relevance reflects if the change over time is meaningful for a patient. A change can be statistically significant (particularly when the sample is sufficiently large), but that doesn't mean the change is also clinically relevant. In my opinion, all PRO results should be interpreted in terms of both a statistical and clinical relevance. | Q5 |
|  | Clinically relevant is a change that has important impact on the patient's quality of life. A difference of 1 in a score can become statistically significant if there are 1000 patients in the trial, but is may have NO clincal relevance at all! | Q17 |
|  | clinical relevance should be based on the real reflection of patients' concerns. Hence, this does not mean only the significant statistical changes in scores | Q22 |
|  | Is the QoL score or the change in the score important to address? Maybe the QoL score between two groups is statistically different but still below the threshold of clinical relevance (not important enough to do something about it). | Q57 |
|  | A difference can be statistically significant (in layman's terms, to large to likely be due to random chance), and yet not clinically relevant (e.g. the difference between the two groups begin compared is to small to be clinically important). Note: If a difference is large enough to mean a definition of clinically relevant, but it is not statistically significant, the difference is likely not clinically relevant. Clinical relevant difference is statistically significant difference that is large enough to be meaningful to patients and physicians. | Q27 |
|  | This is indeed a difficult problem. One generally understands the term so that a difference is clinically relevant if a patient or a clinicians thinks that the score has changed noticeably or significantly (not statistically significantly) for a single patient. However, I think that an intervention has also a clinically relevant effect if only every second patient profits from the intervention in a personally significant way; that is, I think that also half of the "individual" clinically relevant change is clinically meaningful. | Q41 |
| Not applicable | size and scope of results are large enough | Q87 |
|  | Numbers are less important as qualitative data are relevant in PRO | Q88 |
|  | Need to know more about PROs | Q89 |
|  | Whether the difference or change in PRO outcome is also clinically relevant. | Q90 |
|  | The ability to measure and communicate relevant, interpretable, and meaningful changes in symptoms, function, and HRQOL for patients with cancer. | Q91 |
|  | detectable and sustained change | Q92 |

***B. Allocation frequencies of codes and clusters***

| **Cluster (N=5)**  ***Allocations N=104*** | **Codes (N=15)**  ***Allocations N=130*** |
| --- | --- |
| **A Patient value (n=49)** | A.1 Meaningful for patient (n=28) |
|  | A.2 Noticeable for patient (n=17) |
|  | Impact on patient (n=9) |
| **B Practical implications (n=32)** | B.1 Action must be taken (n=18) |
|  | B.2 Importance for clinical practice (n=8) |
|  | B.3 Clinical importance (n=7) |
| **C Reference to external criteria (n= 9)** | C.1 Reference to clinical values (n=5) |
|  | C.2 Reference to objectively measurable criteria (n=4) |
| **D Statistical approach (n=7)** | D.1 Difference to control group (n=4) |
|  | D.2 Statistical parameters (n=3) |
| **E Proxy value (n=7)** | E.1 Meaningful for HCPs or proxies (n=4) |
|  | E.2 Noticeable for HCPs or proxies (n=3) |

***C.* *Interrelation of clusters of Clinical Relevance -*** *the clusters’ relative sizes (number of quotes allocated) and their content-related connections*


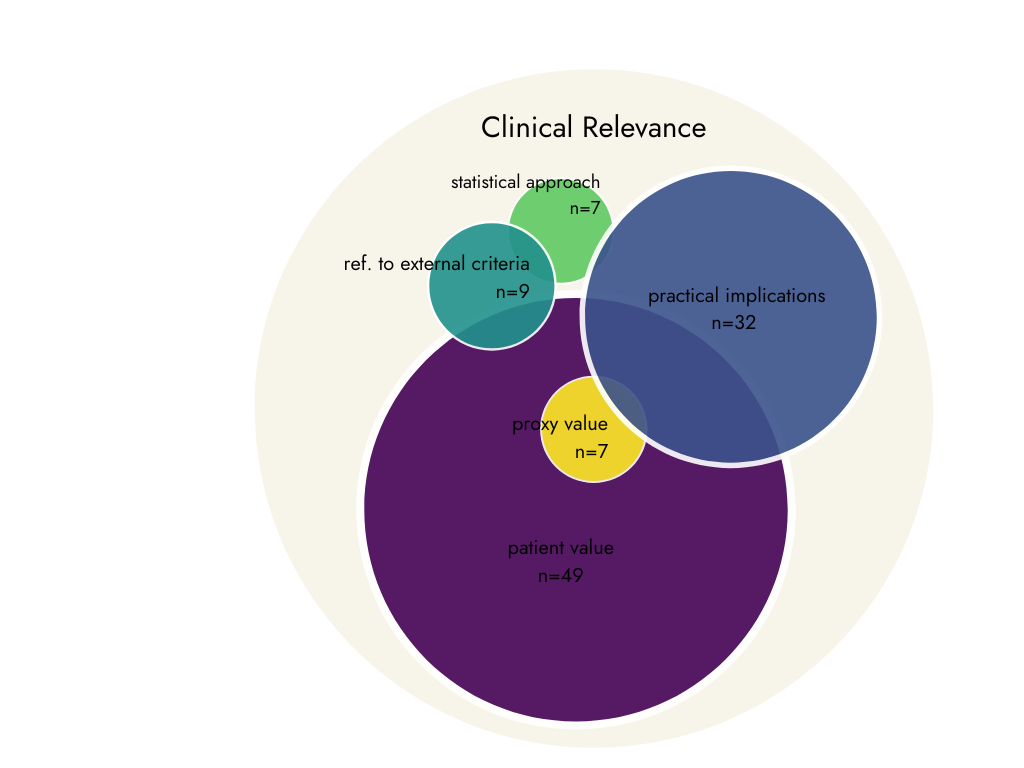


| ***D.* *Overlaps in the allocation of clusters*** | | | | | | |
| --- | --- | --- | --- | --- | --- | --- |
|  |  | Patient value | Practical implications | Reference to external criteria | Statistical approach | Proxy value |
|  |  |  |  |  |  |  |
| Patient value (n=49) | n (joint mentions) |  | 10 | 2 | 0 | 7 |
|  | % of patient value |  | 20.4% | 4.1% | 0.0% | 14.3% |
| Practical  implications | n (joint mentions) | 10 |  | 0 | 1 | 1 |
|  | % of practical implications | 31.1% |  | 0.0% | 3.1% | 3.1% |
| Reference to external criteria | n (joint mentions) | 2 | 0 |  | 1 | 0 |
|  | % of reference external criteria | 22.2% | 0.0% |  | 11.1% | 0.0% |
| Statistical  approach | n (joint mentions) | 0 | 1 | 1 |  | 0 |
|  | % of statistical approach | 0.0% | 14.3% | 14.3% |  | 0.0% |
| Proxy value | n (joint mentions) | 7 | 1 | 0 | 0 |  |
|  | % of proxy value | 100.0% | 14.3% | 0.0% | 0.0% |  |

*green = high percentage of joint cluster mentions; red = no joint cluster mentions (arbitrary threshold)*
